# Ether and Chloroform

**Published:** 1876-05

**Authors:** 


### ARTICLE VIII.

### *Ether and Chloroform.*

Ether is preferred to chloroform as an anæsthetic in the Laboratory of Florence, because a very extensive experience has convinced Dr. Schiff that etherization carried to the last grade of insensibility never becomes dangerous to life as long as the movements of respiration are maintained. But even if the inhalation of ether be still further continued until the respiratory movements cease, or in other words, the appearance of death is complete, death does not actually follow if only, at the moment when the thoracic walls are paralyzed, the inhalation is interrupted in order to begin suddenly a kind of artificial respiration by interropted compression of the thoracic walls themselves. By this measure, the air impregnated with ether passes out of the lungs at every compression, and atmospheric air enters every time that the compression is relaxed, and the air so entering takes up more ether, which is again expelled at the susequeut compression. Thus the quantity of ether in the blood is diminished to such a degree as to be no longer sufficient to embarrass the action of the nervous centre of respiration, and thus breathing begins anew. Chloroform has been often popularly preferred to ether because it acts more rapidly, and its application is more agreeable to the patient who dislikes the smell of ether. But according to Dr. Schiff (whose views in this respect seem to be gaining ground in the present day,) chloroform has a much more extensively paralyzing action than ether, and has moreover —at least in man and other mammiferous animals,—a special influence on the nerves of the heart. Thus it happens, that, when chloroform is carried to such a degree as to weaken considerably the respiratory movements, it is sometimes impossible to restore them, in consequence of the weakness of the heart's action. Dr. Schiff goes so far as to state emphatically that a medical man is responsible for every case of death caused by the use of ether—by which he means that a careful watching of the respiration during the emplooment of this agent may always prevent death; while the effects of chloroform depend partly on individual predisposition, which medical science cannot always detect. —*Med. Times and Gazette.*

